# Iodide of Potassium in Hpdrocephalus

**Published:** 1847-08

**Authors:** 


*Iodide of Potassium in Hydrocephalus.*—Several cases have been reported of late in different medical periodicals, which apparently exemplify striking results from the employment of Iodide of Potassium in hydrocephalus. A case has lately occurred in our practice in which the patient unexpectedly recovered under the use of this remedy. The Editor of the Annalist observes, in view of the evidence offered of its efficacy, that "it would be criminal in future to omit its use in this intractable disease."

